# Reflections of Pro-Poor Growth across Agro-Climatic Zones for Farming and Non-Farming Communities: Evidence from Punjab, Pakistan

**DOI:** 10.3390/ijerph19095516

**Published:** 2022-05-01

**Authors:** Aadil Hameed Shah, Atta Ullah Khan, Liurong Pan, Asad Amin, Abbas Ali Chandio

**Affiliations:** 1 Department of Economics, Government Degree College, Ban Hafiz Jee Mianwali 42230, Pakistan; aadilshah777@gmail.com; 2 Department of Economics, Allama Iqbal Open University, Islamabad 44310, Pakistan; attaullah.khan@aiou.edu.pk; 3 Faculty of Economics and Management, Beibu Gulf University, Qinzhou 535011, China; 4 Postdoctoral Station of Management Science and Engineering, College of Economics and Management, Nanjing University of Aeronautics and Astronautics, Nanjing 210016, China; 5 College of Economics, Sichuan Agricultural University, Chengdu 611130, China; alichandio@sicau.edu.cn

**Keywords:** poverty, inequality, Poverty Equivalent Growth Rate (PEGR), pro-poor growth, agro-climatic zone, farming, non-farming

## Abstract

The basic objective of the existing study was to inspect the triangular association between economic growth, poverty, and income disparity in farming and non-farming communities across agro-climatic zones in Punjab province, Pakistan. The cross-sectional Household Integrated Economic Survey (HIES) data and Poverty Equivalent Growth Rate (PEGR) methodology were applied from 2001–2002 to 2015–2016. Outcomes of the study found that in a short period, 2001–2002 to 2004–2005; 2004–2005 to 2005–2006; 2005–2006 to 2007–2008; 2007–2008 to 2010–2011; 2010–2011 to 2011–2012; 2011–2012 to 2013–2014; and 2013–2014 to 2015–2016, economic growth has presented hybrid (pro-poor and anti-poor) pattern across both communities of all agro-climatic zones of Punjab province in different periods. In the longer period of 2001–2002 to 2015–2016, economic growth has been pro-poor across both communities of all the zones apart from zone III (Cotton-Wheat Punjab); there is an anti-poor pattern of economic growth. Results for the decomposition of change in poverty further indicate that economic growth is a dominant factor in reducing poverty for all investigated zone. Moreover, a positive redistribution component reduces the beneficial impacts of economic growth for the poor more than for non-poor, that ultimately makes economic development patterns anti-poor in zone III. In the present study, we proposed two-fold policy implications. First, improve the living standard of households in each agro-climatic zone by increasing their incomes. Second, develop a precise taxation system that helps to reduce income disparities among upper-pro to lower-income groups.

## 1. Introduction

The instability of economic growth and diverse situations revealed by poverty and inequality indices represent a key concern for developing economies. Economic performance resulting from the diverse masses of the population and the biases among different sub-groups has led the academic community to probe the linkages between economic growth and poverty. Most economies have witnessed below-average economic growth, despite considerable performance growth in the South Asian region over several decades [1,2]. Similarly, the economy of Pakistan has also experienced below-average economic growth and is placed in the category of lower-middle-income countries. In this context, Pakistan has been recently ranked 154th among 189 countries (well below average) in the human development category [3]. Additionally, the importance of diverse reflections on economic achievements at various sub-national levels has been significantly amplified in the light of Millennium Development Goals (MDGs) [4,5].

In the modern age of industrial development, where capital is abundant, developing countries around the globe are suffering from many social, political, and economic challenges. One of the biggest and most severe is poverty. Usually, poverty has been described in many ways, while in the eyes of classical and neo-classical economists, it has two major branches i.e., unidimensional and multidimensional poverty [6]. Classical views are predominantly grounded on the notion of the unidimensional idea that describes the paucity of income (less than $1.25 a day) or consumption expenditure (less than 2350 calories/adult a day in monetary form) that makes individuals or households unable to buy a necessary basket of goods for the gratification of materialistic human wants [7]. Neo-classical views based on Sen’s ‘capability deprivation’ defined poverty comprehensively as a multidimensional phenomenon with a lack of access to fundamentally needed social aspects (health, education, and housing facilities) that are essentially needed for humans to satisfy elementary needs and sustain a blissful life [8,9]. In the recent era, multidimensional poverty is categorized into child poverty, health poverty, energy poverty, and food poverty [10,11]. 

Besides, multidimensional poverty, unidimensional (income poverty) is also broadly expressed under a new idea known as pro-poor growth, which describes the triangular relationship between income disparity, economic growth, and income poverty [12]. Pro-poor growth is defined as ”An economic growth” that is said to be pro-poor if it increases average income and declines income disparities [13]. 

Predominantly, economic growth is expected to reduce poverty significantly; however, economic growth loses importance when inequality increases. Consequently, the growth benefits are accumulated in the hands of a few rich individuals, with little or no benefits for lower-income groups due to the trickle-down effect [1,4,14]. Theoretically, a more comprehensive view of economic growth and poverty reduction linkages can also be obtained through pro-poor growth [15]. In essence, pro-poor growth explains how the poor are affected by growth, how growth benefits are delivered to the poor, and how much payback the poor gain from the growth of an economy [16,17,18,19]. Additionally, refs. [20,21] have demonstrated the effectiveness of examining growth performance in poverty reduction through a tri-lateral association between growth, poverty, and inequality. Most studies [22,23] believe in a strong association between growth, poverty, and inequality. 

For example, ref. [24] indicated that the association between growth, poverty, and inequality is convoluted and suggested that economic growth alone is a poor tool for examining poverty reduction [25]. Meanwhile, refs. [26,27] argued against the notion of inequality-led growth. Similarly, refs. [28,29] found no linkage between growth and inequality. Consequently, the failure of the trickle-down effect and the emergence of biases among the various sectors in developing economies are in stark contrast to earlier literary discussions [30]. 

Finally, a tri-lateral relationship dominates the existing literature that poverty reduction largely relies upon economic growth and income inequality. The association between economic growth, poverty, and inequality is complicated, nonlinear, and path-dependent. According to [31], for the inverted U-hypothesis in the early stages, economic growth exacerbates inequality, leading to inequality reaching its highest level. However, inequality declines as the benefits of growth trickle down to the lower-income quintiles, which ultimately reduce poverty [32,33,34]. 

Similar to other developing nations, the economy of Pakistan has experienced different phases of economic fluctuations and followed various growth-led policies. In this respect, a wide array of literature emphasizes the triangular relationship related to the growth of poverty and inequality throughout history. However, it is unanimously agreed that there was a significant increase in the growth pattern, beginning in the 1960s [1], although this was mainly because of the green revolution in the agriculture sector [35]. Unfortunately, this economic uplift failed to eradicate poverty due to the poor trickle-down of growth benefits to lower-income groups.

Additionally, as demonstrated by the statistics, this economic growth failed to overcome inequality. Growth has become pro-poor at the national level but anti-poor at the regional level [36]. However, the subsequent decades experienced supreme growth and were known as the golden era of Pakistan’s economy. This growth period was auspiciously accompanied by the appropriate trickle-down effects, resulting in pro-poor growth at the national and regional levels. 

However, the 1990s witnessed several shocks (corruption, political instability, and historic droughts). The adverse economic performance severely affected the poor segments of the population, particularly in rural areas. Consequently, this situation affected the poor, with the economy being considered as anti-poor [1,30]. A smaller rise in growth was seen in the 2000s. However, when compared to the 1990s, this economic success had a marginally different impact on the poor due to the proactive government approach. The era of the 2000s is regarded as pro-poor for Pakistan’s economy at national and regional levels [14,37,38].

The literature reveals various techniques to explore the growth, poverty, and inequality nexus through the notion of pro-poor growth. However, the most prominent methods are poverty equivalent growth rate (PEGR), poverty bias growth (PBG), and the pro-poor growth index (PPGI), which depict an appropriate picture of pro-poorness. Moreover, they identify how growth benefits are delivered to the poor and non-poor and how much gain (loss) is faced under the different measures of pro-poor growth. Various authors have frequently employed similar methodologies to probe pro-poor growth; for example, ref. [39] used the PBG index and [40] employed the poverty growth curve (PGC) to examine the growth patterns of poverty. Similarly, ref. [18] measured relative and absolute pro-poor growth using the widely accepted PEGR index. Omer and Jafri [37] examined pro-poor growth in Pakistan over the past four decades using the growth incidence curve (GIC). Authors in [13] estimated pro-poor growth in Pakistan’s sub-sectors (agriculture, commodity-producing, manufacturing, and the service sector) using the PPGI and PEGR indices. Finally, ref. [41] calculated pro-poor growth in absolute and relative terms in the agro-climatic zones of Pakistan from 1998 to 2011 using the PEGR index. 

Although several methods have been employed to examine pro-poor growth at the national and provincial levels in Pakistan, a single study has not been found that explored pro-poor growth among farming and non-farming communities across the agro-climatic zones of the Punjab province in Pakistan over the last one-and-a-half decades. Therefore, this study differentiated from other studies, especially [33], in three ways; initially, it increased the study time length from one decade to fifteen years. Secondly, it included decomposing the change in poverty into growth and redistribution components. Thirdly, it validated the outcomes of PEGR through GIC curves, which have been lacking in the existing literature, especially in the context of agro-climatic zones of Pakistan. 

After the initial phase of detail introduction and literature review, the rest of the study has been categorized into following steps. Section 2 explains the methodology and also presents the data. Section 3 presents the overall outcomes of the study with a detailed discussion of poverty, inequality, and pro-poor growth methodologies. Section 4 concludes the entire paper and also provides various policy implications in light of the empirical findings.

## 2. Methodology

### 2.1. Data Description

For a deep understanding of poverty, growth, and income disparity triangular association between the farming and non-farming community of agro-climatic zones of Punjab province of Pakistan, cross-sectional data of HIES (Household Integrated Economic Survey) for the last fifteen years from 2001–2002 to 2015–2016 has been used, which collected from the globally acknowledged institute of PBS (Pakistan Bureau of Statistics). PBS has done a country-wide survey periodically to collect precise HIES data sets. A well-acknowledged universally putative questionnaire has been used to attain information at the individual and household levels. In contrast, a proportionate random sampling statistical method has been adopted to select the opposite sample size. After completing the data collection process, various PSUs (Primary Sampling Units) and SSUs (Secondary Sampling Units) have been developed to arrange the data. The set of every PSU consists of an enumeration block of urban areas and Mouzas, dehs, and villages of backward or rural areas.

Similarly, SSU has been obtained from PSU, which involves 16 households from the rural region and 12 households from urban regions [42]. All the datasheets have been categorically divided into two different forms. A few sheets are prepared at the household level, where gathered information is common among all individuals residing in the same house. In contrast, most sheets are prepared at the individual level due to varying information from individual to individual.

### 2.2. Spatial Description of Pakistan and Its Agro-Climatic Zones

In the light of [43] study, the agricultural country of Pakistan is geographically located in the South Asian subcontinent between 24–37° N latitude and 61–76° N longitude. The overall land area of Pakistan is 796,096 Km^2^, which is firmly divided into three different segments; the federal area, four provinces (Punjab, Sindh, KPK, and Baluchistan), and the tribal area, FATA. On the bases of residents (area), Punjab (Baluchistan) is the major province. Around 55.63% of the country’s population resides in Punjab, while Baluchistan covers around 44% of the total area [10]. Furthermore, all these provinces are categorized into a couple of regions i.e., urban and rural, administrative divisions, and districts. According to [10], Punjab is sub-categorized into eight main divisions and 37 districts, counting federal area, while Sind, KPK, and Baluchistan are categorically divided into 6,7, and 6 divisions, and 24, 33, and 35 districts, respectively. 

**Figure 1:**
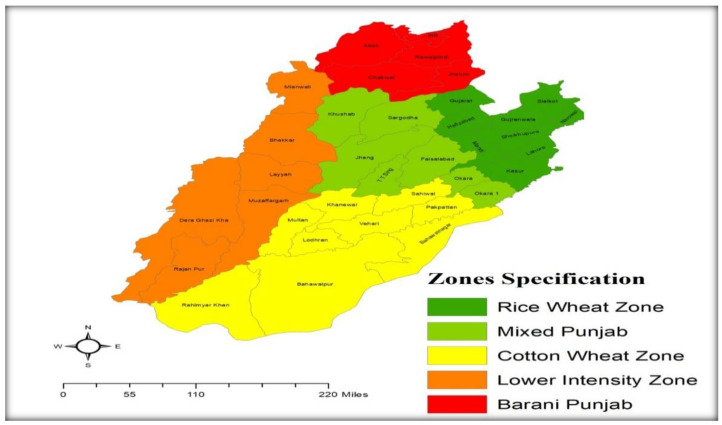
Spatial Map of Agro-Climatic Zones of Province Punjab, Pakistan.

Moreover, ref. [44] categorized the Pakistan economy into a new direction known as agro-climatic zones. According to their study, four provinces, federal areas, and FATA can also be divided into ten dissimilar parts regarded as agro-climatic zones. On the basis of geographical location, Punjab, which is the area of discussion for the current study, is divided into five crucial agro-climatic zones, (I) Rice-Wheat Punjab, (II) Mixed Punjab, (III) Cotton-Wheat Punjab, (IV) Lower Intensity Punjab, and (V) Arid Zone Punjab the detail of these zone has also been specially persented in Figure 1. Furthermore, [3] sub-divided the residences of these zones into farming and non-farming communities. However, the population share of the farming (non-farming) community is around 67.13% (32.87%) of the overall province of Punjab in each data set that is taken under consideration from 2001–2002 to 2015–2016.

The welfare indicator is derived based on households’ per capita consumption/expenditure. As adopted by the Planning Commission of Pakistan, a threshold (monetary value of 2350 calories per adult per day) has been used as a poverty line. Moreover, the given poverty line has been inflated accordingly every year to incorporate the inflation changes through the consumer price index (CPI) at national and sub-national levels [42]. Meanwhile, the welfare indicator of households is calculated by dividing the total expenditure of the adult equivalent household size:

(1) $$Y=\frac{TCE}{AEHS}$$

where *Y* denotes the per capita consumption expenditure of households and *TCE* represents the total consumption expenditure of households. These are estimated by taking the expenses for all purchased and self-produced items, gifts, wages, and salary for consumption, excluding taxes, marriage, fines, and expenses for durable goods. Finally, *AEHS* is the adult equivalent household size, which is calculated based on the nutrient requirements of family members, which has been assigned the weight of 0.8 (1) for individuals less (greater) than 18 years old [45].

### 2.3. Poverty Measurement

The poverty profile in the unidimensional spectrum is measured using the [46] index approach, reflecting the incidence, depth, and severe poverty. This index is generally expressed using a single equation and varying poverty aversion indicator (α) or the weight parameter. Equation (2) below illustrates the general formation of the FGT index:

(2) $$FGT=\frac{1}{n}\overset{q}{\underset{i=1}{\sum }}{\left(\frac{z-y}{z}\right)}^{\alpha }$$

where *n* is the total population, *q* is the number of non-poor households, *y* is per capita consumption expenditure, and *z* is the minimum threshold distinguish between poor and non-poor. Finally, *α* is the weight parameter, which varies from 0 to 2, reflecting poverty’s incidence, depth, and severity. The severe poverty represents inequality between those clustered around the poverty line and the ultra-poor, or those well below the poverty line.

### 2.4. Inequality Measurement

Inequality from the unidimensional perspective is measured using the widely accepted Gini coefficient. The Gini coefficient is defined as “the ratio of the area between the Lorenz curve and the line of equality, the area of the triangle below this line”. Estimates for the Gini coefficient range from 0 to 1 and are specified as 0 ≤ *G* ≤ 1, where “0” explains perfect equality while “1” explains perfect inequality [47]. Mathematically, this measure is described as:

(3) $$\mathrm{Gini}\;\mathrm{coefficient}=1-\overset{n-1}{\underset{i=0}{\sum }}\left(T_{i=1}-T_{I}\right)\left(\delta_{i+1}+\delta_{i}\right)$$

where *T_i_* is the cumulative share of the population and *δ_i_* is the cumulative share of consumption.

### 2.5. Decomposition of Poverty into Growth and Redistribution Components

Initially, [48] presented the concept of decomposing change in poverty into growth, distribution, and residual components between two distinct study periods. The residual or unexplained portion prevails when poverty calculations are not decomposable into mean expenditure and distribution [49]. The decomposition measure has a severe limitation, i.e., the calculated unexplained portion or residual becomes relatively larger and casts doubt on the evaluated outcomes. Kakwani [20] addressed these drawbacks and presented another axiomatic approach. The residual or unexplained portion is replaced with the simple averaging method, decomposing poverty into growth and distribution components. Generally, this measure is expressed as follows:

(4) $$\Delta P=P_{j}-P_{i}=G+D$$

(5) $$G=0.5\left[\left[P\left(\frac{z_{o}}{\upsilon_{j}},L_{i}\right)-P\left(\frac{z_{o}}{\upsilon_{i}},L_{i}\right)\right]+\left[\left(\frac{z_{o}}{\upsilon_{j}},L_{j}\right)-\left(\frac{z_{o}}{\upsilon_{i}},L_{j}\right)\right]\right]$$

(6) $$D=0.5\left[\left[P\left(\frac{z_{o}}{\upsilon_{i}},L_{j}\right)-P\left(\frac{z_{o}}{\upsilon_{i}},L_{i}\right)\right]+\left[\left(\frac{z_{o}}{\upsilon_{j}},L_{j}\right)-\left(\frac{z_{o}}{\upsilon_{i}},L_{i}\right)\right]\right]$$

where Δ*P* is the change in poverty between the terminal and initial period, *G* represents the growth components, *D* represents the distribution components, *P* represents the poverty calculations, subscript *ij* shows the initial and terminal period, *z* is the minimum threshold/poverty line, *υ_ij_* represents the average per capita consumption expenditure in the initial and terminal year, and, finally, *L_ij_* shows the Lorenz curve measure for the periods under consideration.

### 2.6. Systematic Framework for the Assessment of the Poverty Equivalent Growth Rate (PEGR)

Kakwani and Pernia [50] presented a comprehensive pro-poor growth index (PPGI). This measurement explains that growth is pro-poor in a relative and absolute sense if poverty and inequality decline during the growth process. It has also been defined as follows: “growth is pro-poor in both relative and absolute terms if poor receive proportionally more benefits from growth than the non-poor” [18]. The PPGI index is mathematically written as:

(7) $$\phi =\left(\frac{\delta }{\eta }\right)$$

where *φ* is the relative pro-poor growth index, *δ* is the total growth elasticity of poverty, and *η* is the growth elasticity of poverty with a constant inequality effect in a relative sense. It is generally expressed as:

(8) $$\eta =\frac{1}{\theta }\int_{0}^{H}\frac{\partial P}{\partial x}x\left(p\right)dp$$

However, in an absolute sense, φ* = (*δ*/η*), where η* is the growth elasticity of poverty with a constant inequality effect. It is generally derived as follows:

(9) $$\eta^{*}=\frac{\mu }{\theta }\int_{0}^{H}\frac{\partial P}{\partial x}dp$$

The indexing approach is of great importance in estimating pro-poor growth. However, it has a severe shortcoming, i.e., it estimates the growth benefits’ distribution among poor and non-poor without considering the level of real growth rate. To overcome these drawbacks, [12] proposed another, widely accepted, pro-poor growth index (PEGR). The PEGR has an advantage that comprises the real growth rate and explores the degree to which the poor get benefit from growth, as follows:

(10) $$\gamma^{*}=\left(\frac{\delta }{\eta }\right)\gamma =\phi \gamma$$

where *γ* = dLn(µ) is the growth rate of average consumption expenditure, *ϕ* = (*δ*/*η*) is the pro-poor index developed by [50], and γ* is the relative PEGR. 

Thus, outcomes of the PEGR index can be explained as: growth is regarded as strongly pro-poor if γ* > *γ*, but as anti-poor when γ* < *γ*. If 0 < γ* < *γ*; growth is not strictly pro-poor just because severe poverty declined during the growth process, if inequality worsened. Such a situation is also known as the trickle-down process, whereby the poor receive proportionally fewer benefits from growth than the non-poor. Suppose *γ* > 0 and γ* < 0, this situation is regarded as “immiserizing growth; where inequality worsened to a greater extent, which has counterbalanced the beneficial effect of growth and caused the poverty to increase” [51,52]. However, growth is also regarded as strongly pro-poor during a recession period when γ* > 0 and *γ* < 0. Despite negative growth, poverty still declines due to a larger decline in poverty than the negative impact of growth. Similarly, growth is regarded as pro-poor when γ* > *γ*, but both are less than 0; negative growth harms the poor less than the non-poor. Finally, growth is also regarded as anti-poor during a downturn when γ* < *γ* and both are less than 0. Negative growth aggravates poverty, ultimately hurting the poor proportionately to a greater extent than the non-poor. 

## 3. Results and Discussion

**Figure 2:**
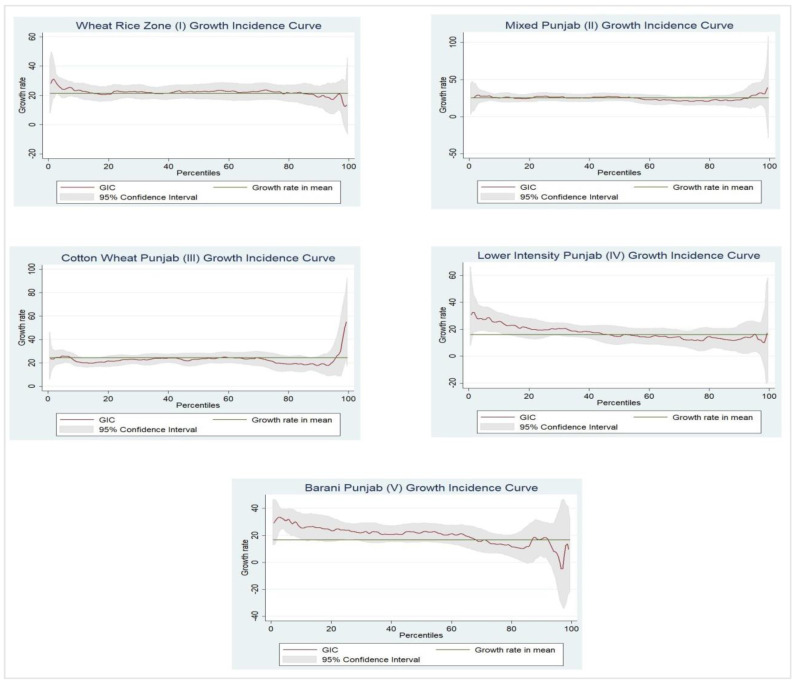
Growth incidence curve for farming communities across all agro_climatic zones in Punjab.

**Figure 3:**
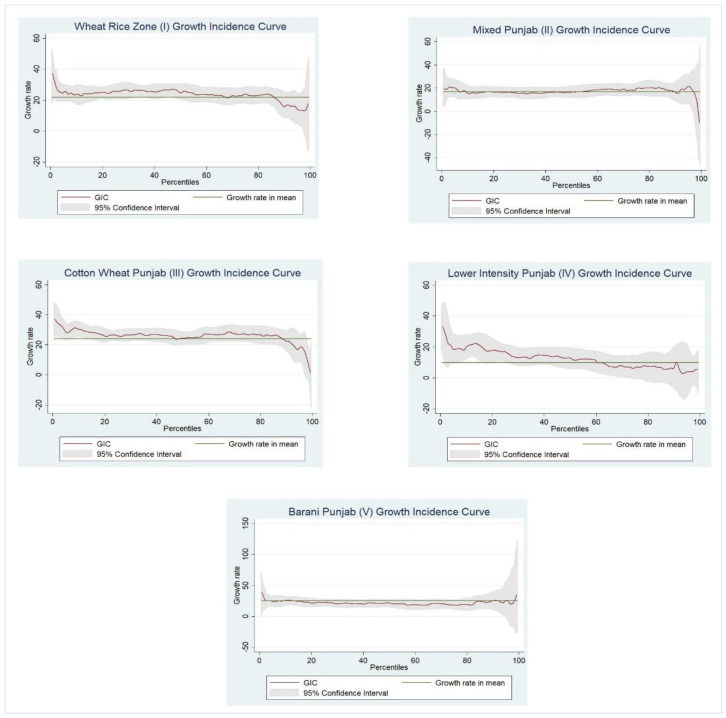
Growth incidence curve for non-farming communities across all agro_climatic zones in Punjab.

The reflections on poverty and inequality among agro-climatic zones for farming and non-farming communities in Punjab (Pakistan) from 2001–2002 to 2015–2016 are presented in Table 1. The findings reveal that the magnitude of poverty in the farming community has significantly declined in this period across all three measures (headcount index, poverty gap index, and squared poverty gap index). The stepwise outcomes of these measures in zones I–V indicate that, respectively, 26.05, 35.71, 45.00, 47.63, and 18.77% of households were poor in 2001–2002, while these measures significantly declined to 2.63, 5.17, 11.72, 18.54, and 1.265%, respectively, in 2015–2016. Similarly, the depth of poverty among households in all zones was estimated as 5.11, 8.05, 10.07, 13.36, and 3.89%, respectively, in 2001–2002, compared to 0.30, 0.86, 1.25, 2.94, and 0.17%, respectively, in 2015–2016. Finally, the severity of poverty is estimated as 1.521, 2.58, 3.166, 5.166, and 1.110% in 2001–2002 among the poor farming community households in all agro-climatic zones of Punjab, while in 2015–2016, the severity reduced considerably (0.078, 0.245, 0.251, 0.685, and 0.025%, respectively). However, such a favorable environment is mainly attribute to the positive growth performance between the two periods [38], which caused a significant decline in poverty among farming community households in all agro-climatic zones in Punjab. The other measures of consumption inequality revealed a mixed trend, i.e., zones II and III showed a marginal increase in inequality. In contrast, zones I, IV, and V showed a marginal decline in inequality between the two periods [14]. Apart from the overall description, zone I (with a short period, primarily one- or two-years gap, such as 2001–2002 to 2004–2005 and 2004–2005 to 2005–2006 etc.) showed a declining trend in poverty; all other agro-climatic zones showed a fluctuating trend for poverty among the farming communities. On the other hand, the trend for inequality measures in such cases continued to fluctuate for all the zones. In the case of inequality over a short period (smaller time gap of two years, such as 2001–2002 to 2004–2005 and 2004–2005 to 2005–2006 etc.), zones V (I) and II (IV) were the most (least) affected in terms of inequality in 2001–2002 and 2015–2016, respectively. Growth incidence curve for farming communities across all agro-climatic zones in Punjab are shown in Figure 2 and growth incidence curve for non-farming communities across all agro-climatic zones are shown in Punjab Figure 3.

The outcomes for non-farming communities in all agro_climatic zones showed largely similar trends for poverty and inequality to those for the farming communities between 2001–2002 and 2015–2016. For example, in zones I–V, around 47.56, 45.16, 60.60, 49.73, and 22.09%, respectively, of households were poor in 2001–2002, declining to 11.86, 18.15, 17.03, 21.80, and 2.40% in 2015–2016 [38]. Similarly, estimates the depth of poverty among households in all zones were 11.40, 10.91, 17.05, 13.42, and 4.43%, respectively, in 2001–2002, decreasing to 1.65, 3.23, 2.26, 3.82, and 0.30% in 2015–2016. Finally, the severity of poverty among the non-farming community across agro-climatic zones was estimated as 3.90, 3.81, 6.43, 5.07, and 1.31%, respectively, in 2001–2002, decreasing to 0.34, 0.86, 0.48, 0.98, and 0.10% in 2015–2016. However, this favorable situation is main attribute to growth-oriented interventions of the government [14,23,47]. 

At the same time, the estimated outcomes for inequality reveal that, between 2001–2002 and 2015–2016, in zones I, III, and IV, a slight decline occurred (25.60 to 24.50%, 24.40 to 22.50%, and 27.20 to 24.30%, respectively). However, in zones II and V, inequality marginally increased (24.10 to 26.80%, 26.40 to 28.10%, respectively). Contrary to the overall scenario, for a short period, i.e., a gap of one or two years, such as 2001–2002 to 2004–2005 and 2004–2005 to 2005–2006, etc., both poverty and inequality portrayed fluctuating trends across all zones for non-farming communities throughout the study period [41]. 

The comparative measures across all zones for non-farming communities show that households in zones III (IV) were most affected, while households in zone V (II) were least affected in terms of poverty (inequality) in 2001–2002 [36,53]. Similarly, outcomes for 2015–2016 exhibit that households in zones IV (V) were the most affected and households in zones V (III) were the least affected in terms of poverty (inequality) [38].

Table 2 presents the actual growth rates of consumption expenditure/adult equivalent and PEGR among the farming and non-farming communities across agro-climatic zones in the Punjab province. The relative measure of PEGR for the farming community reveals the strongly pro-poor growth in zones I, II, IV, and V. For example, for γ* > *γ* (during the growth process), not only did poverty reduction occur, but also poor communities enjoyed proportionally higher benefits of growth than the non-poor [12]. However, in zone III, where growth was regarded as anti-poor, i.e., 0 < γ* < *γ* (during the growth process), inequality decreased significantly, which mitigated the beneficial impact of growth. Explains why the poor enjoyed proportionally fewer benefits of growth than the non-poor [20]. On the other hand, between 2001–2002 and 2015–2016, the PEGR for the depth of poverty among the farming communities in zones I, IV, and V was greater than the actual growth rate of consumption expenditure/adult equivalent, and the PEGR for the severity of poverty measure. Thus, it is evident that growth among the farming communities in zones I, IV, and V had a more beneficial impact on those clustered around the poverty line than on the poor who were below the poverty line. Conversely, in zones II and III, the PEGR for the depth and severity of poverty was less than the actual growth rates, meaning that the impact of growth in both zones was not beneficial for the ultra-poor or the vulnerable poor [50]. 

Similarly, for the non-farming communities, the value of relative PEGR was greater than the actual growth rate of consumption expenditure/adult equivalent (γ* > *γ*) across all the agro-climatic zones of Punjab. This situation reduced poverty, and poor households enjoyed higher growth benefits than the non-poor [38]. On the other hand, regarding the depth and severity of poverty in zones I and II, the PEGR remained smaller than the actual growth rate (γ* < *γ*). Growth impacts in both zones were not beneficial for the ultra-poor and those around the poverty line [18]. Meanwhile, the PEGR for the depth of poverty in zones III, IV, and V zone was greater than the actual growth rate; thus, the poor around the poverty line enjoyed more benefits than the ultra-poor.

Table 3 and Table 4 depict the short-run scenario for the actual growth rate in consumption expenditure/adult equivalent and relative PEGR among the farming and non-farming communities. Between 2001–2002 and 2004–2005, a positive actual growth rate prevailed for farming and non-farming communities across all agro-climatic zones. Thus, this shows the significant improvement in household per capita consumption expenditure over time [23,24]. In comparison, PEGR outcomes indicate that the growth pattern was pro-poor for farming communities in zones I and V, unlike zones II, III, and IV, where growth was anti-poor. Such a situation exists because, during the growth process, inequality worsened to a greater extent and outweighed the positive impact of growth [23,41,47]. However, regarding the depth and severity of poverty, apart from zone V, the PEGR value remained less than the actual growth rate. This indicates that growth was not beneficial for both the ultra-poor and vulnerable poor segments [14].

The PEGR measures reflect that growth was pro-poor for zones I, II, and V in the non-farming communities, while growth was anti-poor for zones III and IV. Therefore, inequality worsened, outweighing the positive impact of growth [37]. Conversely, the depth and severity of poverty exhibited pro-poor growth in zone I and V, which means that the ultra-poor obtained more growth benefits than those around the poverty line [16]. On the other hand, in zones II, III, and IV, the PEGR was less than the actual growth rate, highlighting the negative impact on the ultra-poor and slightly poor [38].

Table 4 depicts the overall actual growth rate as pro-poor, with the PEGR measure presenting pro-poor growth across all zones for farming and non-farming communities. Similarly, the PEGR for the depth and severity of poverty (for pro-poor growth) was greater than the actual growth rate, which shows that growth remained highly beneficial for the ultra-poor and those around the poverty line [38]. On the contrary, a positive actual growth rate for the PEGR represents an anti-poor growth pattern. Inequality mitigates the beneficial impact of growth for the poor during the growth process and decreases the poverty index [13,26,34]. Similarly, the PEGR for the depth and severity of poverty (representing anti-poor growth) shows that the depth and severity of PEGR were less than the actual growth rate, demonstrating the inverse impact of growth both for the ultra-poor and the vulnerable poor. 

Moreover, the positive actual growth rates in Table 3 show negative actual growth rates among farming and non-farming communities across all agro-climatic zones. Table 4 reveals that the actual negative growth, in the case of the PEGR representing pro-poor growth, shows that the PEGR is less negative than actual growth. The growth is pro-poor, and thus, during the recession, the poor are less affected than the non-poor. In the case of the PEGR representing anti-poor growth, the PEGR is more negative than the actual growth rate [13,53]. Hence, in this situation, the downfall hurts the poor more than the non-poor. Similarly, the PEGR for the depth and severity of poverty shows that, if the value of PEGR represents pro-poor growth, the downturn favors the ultra-poor and those clustered around the poverty line because they are less affected. At the same time, if the PEGR represents anti-poor growth, the situation is completely reversed [27]. 

The decomposition of the change in poverty into growth and redistribution components has been explained using [20] methodology in Table 5. Results reveal that, from 2001–2002 to 2015–2016, poverty declined across all three measures for farming communities in all zones, i.e., headcount index, depth of poverty, and severity of poverty. The results show that growth has remained the dominant factor, playing a crucial role in reducing poverty. The importance of redistribution components can never be ignored as they also contribute considerably to reducing poverty [50]. On the contrary, few measures found the redistribution to be positive, but poverty was still reduced due to the leading role of economic growth. However, the beneficial impacts of growth were reduced for the poor when compared to the non-poor. The estimated measures show that poverty reduction occurred across zones I–V (23.40, 30.53, 33.30, 29.09, and 17.50%, respectively) between 2001–2002, 2015–2016. The contribution of economic growth in reducing poverty was approximated as 21.28, 30.02, 35.64, 27.63, and 11.82%, while redistribution components contributed around 2.12, 0.51, 2.34, 1.46, and 5.68%, respectively. Although the redistribution components were positive in zone III, poverty declined due to the dominant growth contribution [12,34]. However, this affected the pro-poor growth situation and made it anti-poor, which reduced the growth benefits for the deprived segments of society. In line with the headcount index, a more or less similar trend of change in poverty, growth, and redistribution components was noted for the depth and severity of poverty.

Similarly, the outcomes for the non-farming communities showed that in zones I–V, a poverty reduction was found (approximately 35.40, 27.00, 43.57, 27.93, and 19.70%, respectively). The magnitude of poverty reduction was estimated at approximately 31.63, 26.95, 40.94, 19.61, and 17.09%, respectively, which contributed to the growth components. Meanwhile, the contribution towards redistribution components was estimated as 4.07, 0.05, 2.63, 8.32, and 2.61%, respectively. However, growth was pro-poor for the headcount index across all zones because both growth and redistribution components were negative and significantly reduced poverty [48]. For the headcount index, a more or less similar trend in the change in poverty, growth, and redistribution components was noted for the depth and severity of poverty among the non-farming communities.

## 4. Conclusions

The basic objective of the existing study was to triangulate reconnaissance associations between growth, poverty, and inequality through poverty equivalent growth rate (PEGR) methodology developed by [10] in agro-climatic zones of the Punjab province of Pakistan, by using cross-sectional data of HIES (Household Integrated Economic Survey) from 2001–2002 to 2015–2016. The results revealed that, between 2001–2002 and 2015–2016, there was a significant decline in poverty for both communities in all zones. However, in the case of a short period (mostly one- or two-years gaps such as 2001–2002 to 2004–2005; 2004–2005 to 2005–2006; 2005–2006 to 2007–2008, etc.), the poverty profile showed a fluctuating trend of positive growth, apt trickle-down of growth benefit toward lower-income quintile, and reduction of income inequality, leading to a decline in poverty. In contrast, negative growth worsened the income distribution pattern, thus increasing poverty. Similarly, the situation regarding pro-poor growth for a longer period of 15 years from 2001–2002 to 2015–2016 shows that, apart from zone III (Cotton-Wheat Punjab), growth was pro-poor for both communities across all zones. The PEGR value for the depth of poverty for the Wheat-Rice zone (I), Lower Intensity Punjab (IV), Barani Punjab (V) (for farming communities) and Cotton-Wheat Punjab (III), Lower Intensity Punjab (IV), and Barani Punjab (V) (for non-farming communities) were greater than the actual growth rate as well as the severity of poverty measure, which means the poor that are clustered around the poverty line are receiving more growth benefits as compared to ultra-poor. On the contrary, the PEGR for the depth and severity of poverty for mixed Punjab (II) and Cotton-Wheat Punjab (III) (in the farming communities) and Wheat-Rice zone (I) and mixed Punjab (II) (in the non-farming communities) was less than the actual growth rate, which means for these zones, growth did not benefit the poor clustered around the poverty line as well as ultra-poor. 

Similarly, in the case of a short period, the PEGR showed a mixed trend across all three poverty measures among the farming and non-farming communities. As shown in Table 5, PEGR values are depicted as “Y” for both communities, and all three poverty measures represent a pro-poor growth pattern. In contrast, values depicted as “N” represent an anti-poor growth pattern. Finally, the decomposition of the change in poverty into growth and redistribution components shows that growth is the dominant factor, playing a significant role in reducing poverty and contributing considerably to overcoming poverty. Although the redistribution component was positive for a few measures such as the farming community’s Cotton-Wheat zone (III), economic growth still mitigated the extent of poverty. In the case of pro-poorness, such positive redistribution components reduce the beneficial impacts of economic growth. If it dominates, it makes the pattern of growth anti-poor in nature. 

### 4.1. Policy Recommendations

This study concludes that economic factors (growth and inequality) are important pillars in reducing poverty in both communities and across all the agro-climatic zones of Punjab, Pakistan. Therefore, the government may adopt a two-fold policy as per policy concerns. First, it should improve the living standard of households in each agro-climatic zone by increasing their incomes. The consumption expenditures of these households may thus rise, ultimately boosting economic growth. Second, the government should develop a precise taxation system that helps to reduce income disparities among upper- to lower-income groups. However, collective improvements in both (growth and inequality) measure lead to an increase in the PEGR, which ultimately benefits both the poor (clustered around the poverty line and ultra-poor) to a great extent and causes a reduction in poverty in the study area. 

### 4.2. Limitation of the Research

Initially, the current study was limited to the agro-climatic zones of the Punjab province; however, in the future, it must be extracted to the agro-climatic zones of the overall country. A zonal description of pro-poor growth has been presented; however, in the future, spatial description of pro-poor growth can also be conducted, which estimates pro-poor growth spatially, not at the zonal level, but also in the future at the district level as well. Finally, the time length of the current study was 1.5 decade. However, with data availability, this study can be stretched to two decades in the near future. 

## Figures and Tables

**Table 1:** Poverty and inequality across agro-climatic zones in Punjab.

Zone	Index	2001–2002	2004–2005	2005–2006	2007–2008	2010–2011	2011–2012	2013–2014	2015–2016
*Farming Community*									
I	P_o_	26.05	15.81	10.80	7.62	6.30	3.14	2.61	2.63
	P_1_	5.11	2.53	1.77	1.10	0.86	0.24	0.37	0.30
	P_2_	1.521	0.629	0.471	0.266	0.181	0.026	0.100	0.078
	InEquality	0.245	0.242	0.264	0.274	0.220	0.223	0.255	0.239
II	P_o_	35.71	24.50	17.45	8.33	9.41	4.07	3.40	5.17
	P_1_	8.05	4.89	2.39	1.16	1.53	0.43	0.51	0.86
	P_2_	2.58	1.52	0.569	0.294	0.391	0.061	0.122	0.245
	InEquality	0.246	30.35	0.258	0.273	0.270	0.222	0.221	0.284
III	P_o_	45.00	40.42	20.21	23.34	16.45	13.95	10.44	11.72
	P_1_	10.07	12.25	4.16	3.75	2.68	1.85	1.51	1.25
	P_2_	3.166	5.27	1.351	0.971	0.715	0.417	0.389	0.251
	InEquality	0.257	0.387	0.261	0.275	0.275	0.251	0.240	0.270
IV	P_o_	47.63	36.70	25.43	29.70	21.32	19.51	24.19	18.54
	P_1_	13.36	10.40	4.27	6.35	3.44	2.67	4.59	2.94
	P_2_	5.160	4.180	1.055	1.861	0.775	0.581	1.250	0.685
	InEquality	0.263	0.334	0.226	0.235	0.225	0.209	0.254	0.228
V	P_o_	18.77	3.08	4.59	1.94	4.45	3.16	2.15	1.265
	P_1_	3.89	0.35	0.83	0.36	0.56	0.19	0.21	0.17
	P_2_	1.110	0.065	0.209	0.135	0.109	0.025	0.034	0.025
	InEquality	0.285	0.260	0.231	0.234	0.239	0.226	0.258	0.256
***Non-Farming Community***									
I	P_o_	47.56	20.04	25.78	11.75	10.48	9.88	4.81	11.86
	P_1_	11.40	3.57	4.70	1.80	1.55	1.42	0.58	1.65
	P_2_	3.902	1.066	1.290	0.404	0.356	0.291	0.112	0.335
	InEquality	0.256	0.222	0.230	0.268	0.231	0.227	0.240	0.245
II	P_o_	45.16	34.31	33.96	19.41	12.77	12.62	9.09	18.15
	P_1_	10.91	7.33	5.83	3.27	2.02	1.54	0.93	3.23
	P_2_	3.810	2.36	1.506	0.939	0.490	0.345	0.165	0.858
	InEquality	0.241	0.280	0.219	0.233	0.259	0.264	0.238	0.268
III	P_o_	60.60	42.28	30.30	35.30	23.50	22.22	22.00	17.03
	P_1_	17.05	13.08	6.86	7.30	3.90	3.80	3.67	2.26
	P_2_	6.43	5.51	2.279	2.060	0.990	0.931	0.905	0.484
	InEquality	0.244	0.358	0.281	0.243	0.232	0.233	0.244	0.225
IV	P_o_	49.73	34.59	32.74	37.40	30.69	35.77	28.81	21.80
	P_1_	13.42	9.12	6.17	7.32	5.72	6.95	5.18	3.82
	P_2_	5.07	3.564	1.690	2.156	1.574	1.856	1.405	0.981
	InEquality	0.272	0.311	0.228	0.276	0.252	0.225	0.268	0.243
V	P_o_	22.09	7.33	2.25	1.70	6.91	4.09	1.83	2.40
	P_1_	4.43	0.50	0.30	0.20	0.76	0.58	0.122	0.30
	P_2_	1.31	0.053	0.045	0.006	0.185	0.155	0.009	0.100
	InEquality	0.264	0.248	0.211	0.234	0.248	0.263	0.296	0.281

Notes: P_o_ = headcount Index; P_1_ = poverty gap index; P_2_ = squared poverty gap index; InEquality = Gini-coefficient measure.

**Table 2:** PEGR in agro-climatic zones of Punjab from 2001–2002 to 2015–2016.

Zone	Index	Farming Communities			Non-Farming Communities		
		*γ*	φ*	γ*	*γ*	φ*	γ*
I	P_o_	47.57	1.106	52.66	48.71	1.042	50.75
	P_1_	47.57	1.035	49.24	48.71	0.986	48.03
	P_2_	47.57	0.997	47.43	48.71	0.983	47.88
II	P_o_	56.86	1.053	59.89	36.66	1.001	36.70
	P_1_	56.86	0.971	55.21	36.66	0.910	33.36
	P_2_	56.86	0.944	53.68	36.66	0.900	33.00
III	P_o_	55.04	0.917	50.47	53.54	1.062	56.85
	P_1_	55.04	0.951	52.34	53.54	1.011	54.13
	P_2_	55.04	0.955	52.60	53.54	1.002	53.65
IV	P_o_	34.43	1.159	39.90	20.53	1.685	34.60
	P_1_	34.43	1.115	38.40	20.53	1.344	27.60
	P_2_	34.43	1.074	36.98	20.53	1.249	25.64
V	P_o_	36.33	1.429	51.92	57.61	1.011	58.24
	P_1_	36.33	1.122	40.76	57.61	0.995	57.32
	P_2_	36.33	1.039	37.75	57.61	0.957	55.13

Notes: P_o_ = headcount Index; P_1_ = poverty gap index; P_2_ = squared poverty gap index; *γ* = growth rate; φ* = relative pro-poor growth index; γ* = relative PEGR.

**Table 3:** Actual growth rate of consumption expenditure across agro-climatic zones in the farming and non-farming communities.

Year	Sector	I	II	III	IV	V
2001–2002 to 2004–2005	Farming	14.68	36.28	27.25	26.12	18.13
	Non-farming	21.01	20.38	48.16	23.28	27.57
2004–2005 to 2005–2006	Farming	10.26	–8.81	1.40	–8.09	–1.43
	Non-farming	–4.17	–11.44	–1.29	–12.46	–0.11
2005–2006 to 2007–2008	Farming	8.68	17.29	–2.13	–3.44	4.93
	Non-farming	31.05	20.87	–13.46	–2.46	–0.87
2007–2008 to 2010–2011	Farming	–4.71	0.24	14.14	12.47	–6.76
	Non-farming	–10.56	12.13	13.21	8.27	–1.11
2010–2011 to 2011–2012	Farming	10.63	4.86	–0.99	2.10	2.67
	Non-farming	4.70	2.57	2.90	–8.92	10.07
2011–2012 to 2013–2014	Farming	13.80	2.65	2.16	3.47	15.07
	Non-farming	15.85	2.23	–0.27	16.27	15.58
2013–2014 to 2015–2016	Farming	–2.67	6.79	14.13	10.32	6.79
	Non-farming	–2.02	–2.20	14.21	8.53	5.65

**Table 4:** Relative PEGR for farming and non-farming communities across agro-climatic zones.

Year	Sector	I	II	III	IV	V
2001–2002 to 2004–2005	Farming	Y (N) [N]	N (N) [N]	N (N) (N)	N (N) [N]	Y (Y) [Y]
	Non-farming	Y (Y) [Y]	N (N) [N]	Y (N) [N]	N (N) [N]	Y (Y) [Y]
2004–2005 to 2005–2006	Farming	N (N) [N]	Y (Y) [Y]	Y (Y) [Y]	Y (Y) [Y]	N (N) [N]
	Non-farming	N (N) [N]	Y (Y) [Y]	Y (Y) [Y]	Y (Y) [Y]	Y (Y) [Y]
2005–2006 to 2007–2008	Farming	N (N) [Y]	N (N) [N]	N (Y) [Y]	N (N) [N]	Y (Y) [Y]
	Non-farming	N (N) [N]	N (N) [N]	Y (Y) [Y]	N (N) [N]	Y (Y) [Y]
2007–2008 to 2010–2011	Farming	Y (Y) [Y]	N (N) [N]	N (N) [N]	N (N) [N]	N (N) [N]
	Non-farming	Y (Y) [Y]	N (N) [N]	N (N) [N]	N (N) [N]	N (N) [N]
2010–2011 to 2011–2012	Farming	Y (Y) [Y]	Y (Y) [Y]	Y (Y) [Y]	Y (Y) [Y]	Y (Y) [Y]
	Non-farming	N (N) [N]	N (N) [Y]	N (N) [N]	Y (Y) [Y]	N (N) [N]
2011–2012 to 2013–2014	Farming	N (N) [N]	N (N) [N]	Y (Y) [N]	N (N) [N]	N (N) [N]
	Non-farming	N (N) [N]	Y (Y) [Y]	Y (Y) [Y]	N (N) [N]	Y (Y) [Y]
2013–2014 to 2015–2016	Farming	Y (Y) [Y]	Y (N) [N]	N (Y) [Y]	Y (Y) [Y]	Y (Y) [Y]
	Non-farming	N (Y) [Y]	N (N) [N]	Y (Y) [Y]	Y (Y) [Y]	Y (N) (N)

Notes: Y = Yes (representing pro-poor growth); N = No (representing anti-poor growth); the depth of poverty is presented in parentheses ( ) and the severity of poverty is presented in square brackets [].

**Table 5:** Decomposition of the change in poverty into growth and redistribution components.

		Farming Communities			Non-Farming Communities		
Zone	Index	Δ*P*	GC	RC	Δ*P*	GC	RC
I	P_o_	−23.40	−21.28	−2.12	−35.70	−31.63	−4.07
	P_1_	−4.81	−4.31	−0.50	−9.74	−8.47	−1.27
	P_2_	−1.44	−1.22	−0.22	−3.57	−3.02	−0.55
II	P_o_	−30.53	−30.02	−0.51	−27.00	−26.95	−0.05
	P_1_	−7.20	−6.95	−0.25	−7.68	−7.71	0.03
	P_2_	−2.34	−2.25	−0.09	−2.95	−2.86	−0.09
III	P_o_	−33.30	−35.64	2.34	−43.57	−40.94	−2.63
	P_1_	−8.81	−9.28	0.47	−14.80	−13.08	−1.72
	P_2_	−2.91	−3.11	0.20	−5.94	−5.03	−0.90
IV	P_o_	−29.09	−27.63	−1.46	−27.93	−19.61	−8.32
	P_1_	−10.43	−7.78	−2.65	−9.61	−5.56	−4.05
	P_2_	−4.47	−2.98	−1.49	−4.09	−2.07	−2.02
V	P_o_	−17.50	−11.82	−5.68	−19.70	−17.09	−2.61
	P_1_	−3.72	−2.23	−1.49	−4.13	−4.23	−0.10
	P_2_	−1.08	−0.64	−0.45	−1.21	−1.20	−0.01

Notes: P_o_ = headcount Index; P_1_ = poverty gap index; P_2_ = squared poverty gap index; Δ*P* = change in poverty; GC = growth components; RC = redistribution components.

## Data Availability

The dataset used in this research are available upon request from the corresponding author. The data are not publicly available due to restrictions i.e., privacy or ethical.
